# Supplementary material for: Increased Expression of Anaphylatoxin C5a-Receptor-1 in Neutrophils and Natural Killer Cells of Preterm Infants
Source: Int J Mol Sci. 2023 Jun 19;24(12):10321. doi: 10.3390/ijms241210321 (PMC10299388; doi:10.3390/ijms241210321)
Supplement: Supplementary file 1 [file ijms-24-10321-s001.zip › Supplementary Figure S3.pdf]

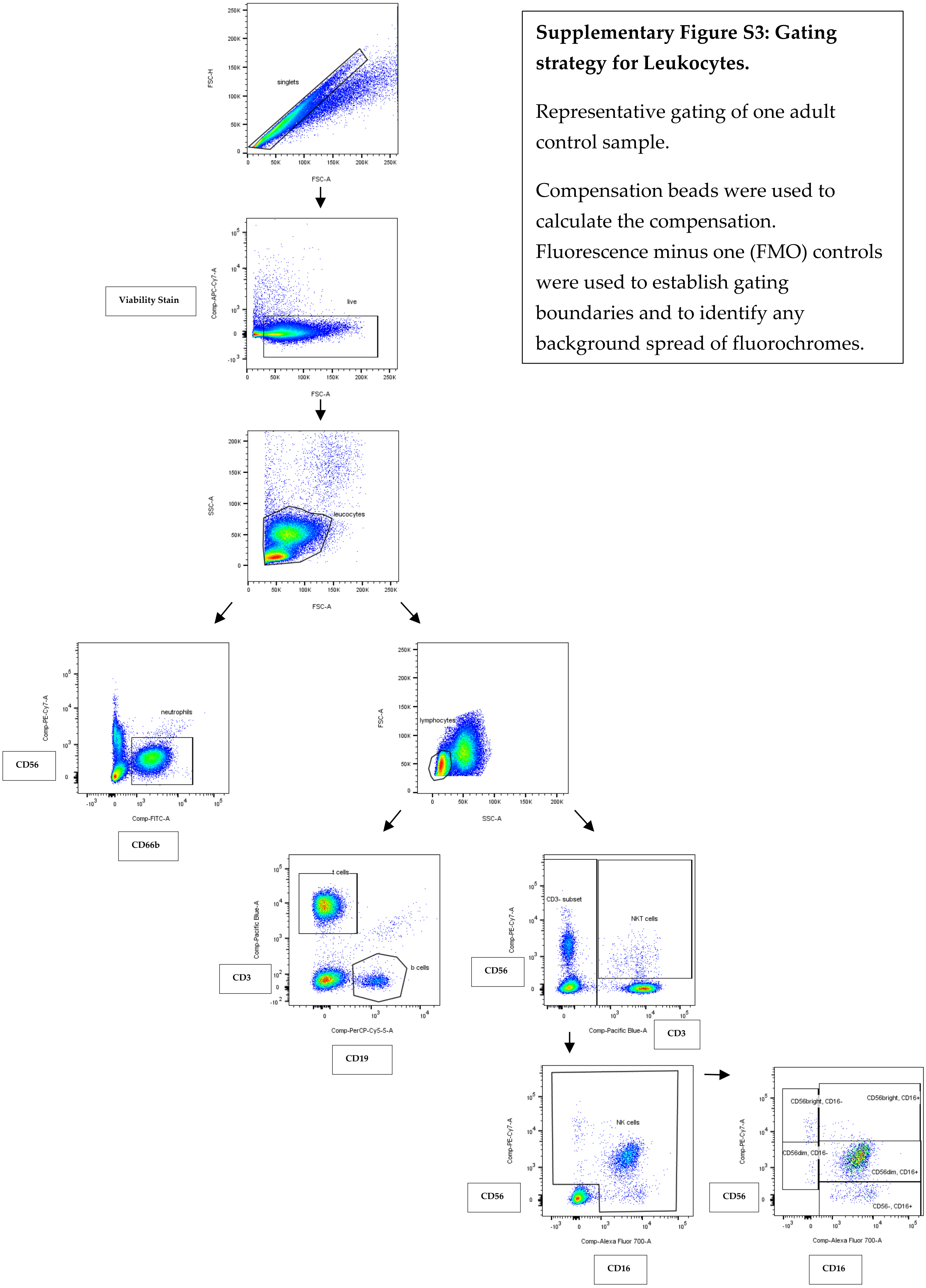

Supplementary Figure S3: Gating strategy for Leukocytes.

Representative gating of one adult control sample.

Compensation beads were used to calculate the compensation. Fluorescence minus one (FMO) controls were used to establish gating boundaries and to identify any background spread of fluorochromes.
