# Supplementary material for: Increased Expression of Anaphylatoxin C5a-Receptor-1 in Neutrophils and Natural Killer Cells of Preterm Infants
Source: Int J Mol Sci. 2023 Jun 19;24(12):10321. doi: 10.3390/ijms241210321 (PMC10299388; doi:10.3390/ijms241210321)
Supplement: Supplementary file 1 [file ijms-24-10321-s001.zip › Supplementary Figures S1 and S2.pdf]

International Journal of
*Molecular Sciences*

*Article*

# Increased Expression of Anaphylatoxin C5a-Receptor-1 in Neutrophils and Natural Killer Cells of Pre-term Infants

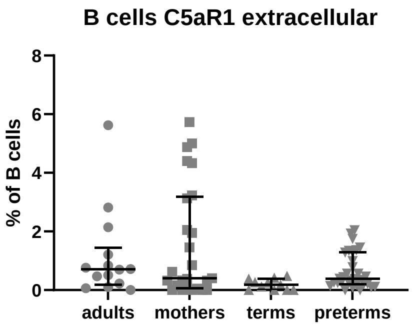


(**a**)

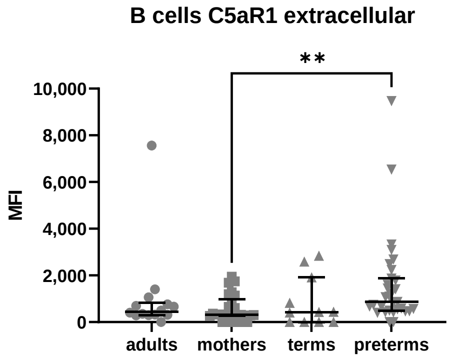


(**b**)

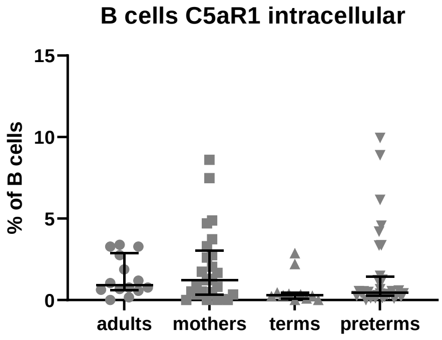


(c)

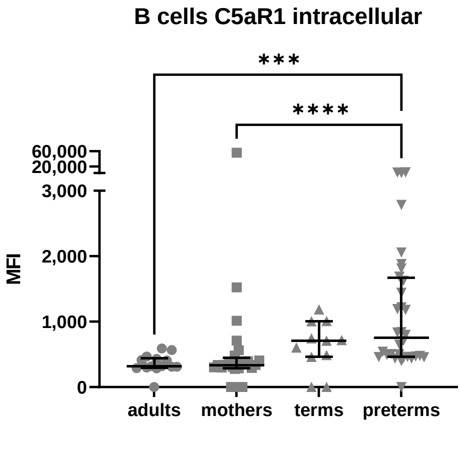


(d)

**Supplementary Figure S1.** Expression of C5aR1 in B cells from adults, mothers, term and preterm infants assessed by flow cytometry: (**a**) Frequencies of extracellular C5aR1-positive B cells; (**b**) Mean fluorescence intensity (MFI) of extracellular C5aR1 on B cells; (**c**) Frequencies of intracellular C5aR1-positive B cells; (**d**) Mean fluorescence intensity (MFI) of intracellular C5aR1 in B cells. Kruskal-Wallis-Test, Tukey´s multiple comparisons, **= $p < 0.01$, ***= $p < 0.001$, **** = $p < 0.0001$, bars indicate Median and interquartile range

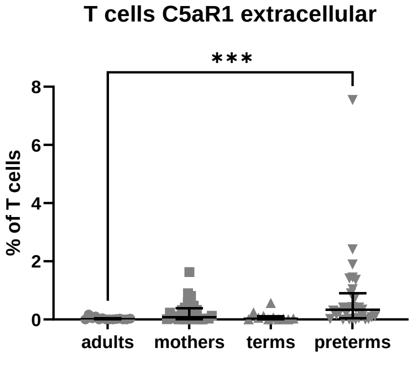


(**a**)

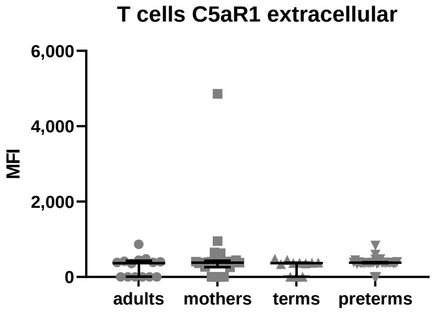


(**b**)

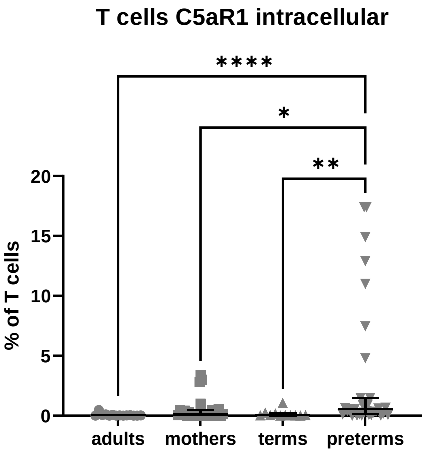


(c)

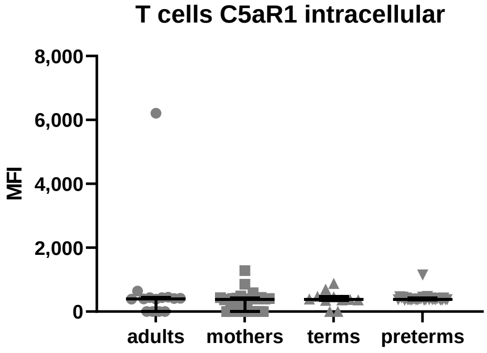


(d)

**Supplementary Figure S2.** Expression of C5aR1 in T cells from adults, mothers, term and preterm infants assessed by flow cytometry: (**a**) Frequencies of extracellular C5aR1-positive T cells; (**b**) Mean fluorescence intensity (MFI) of extracellular C5aR1 on T cells; (**c**) Frequencies of intracellular C5aR1-positive T cells; (**d**) Mean fluorescence intensity (MFI) of intracellular C5aR1 in T cells. Kruskal-Wallis-Test, Tukey´s multiple comparisons, *= $p < 0.05$, **= $p < 0.01$, ***= $p < 0.001$, **** = $p < 0.0001$, bars indicate Median and interquartile range
